# Patrolling Monocytes Watch Over Relapse

**DOI:** 10.1097/HS9.0000000000000451

**Published:** 2020-07-21

**Authors:** Melania Tesio

**Affiliations:** Laboratory of Onco-hematology, Institut Necker Enfants Malades (INEM), Institut national de la Recherche Médicale (INSERM) U1151, Paris, France

The latest years greatly enriched our knowledge of monocytes biology. First, the phenotypic, functional and molecular heterogeneity of this cell population has been largely investigated, revealing the existence of multiple subsets displaying distinct functions in response to different stimuli. Second, monocytes have emerged as important regulators of cancer development and progression, with different subsets playing opposing roles in these processes. Yet, so far, the contribution of monocytes to tumor development appeared to be limited to solid tumors. Nevertheless, monocytes/macrophages populations are important components of bone marrow stem cells niches, and during physiological conditions, they regulate normal hematopoietic stem cells retention and maintenance.^1^ Their role in leukemia development, however, remains unclear. In a recent *Cancer Cell* paper, Witkowski et al fill some gaps in this scenario, demonstrating that non-classical monocytes play an essential role in the pathogenesis of B-cell acute lymphoblastic leukemia (B-ALL),^2^ an hematological cancer whereby B-cell progenitors accumulate in the bone marrow (BM).

By combining single cells RNA sequencing and CITE sequencing, the authors compared the bone marrow composition of 4 healthy bone marrow and seven primary B-ALL encompassing 2 distinct genetic lesions (Ph+ and ETV6/RUNX1). These analyses revealed that two distinct myeloid clusters were significantly changed in the BM of B-ALL patients as compared to the healthy BM samples. The first one, corresponding to so called classical monocytes (CD14^+^CD16^−^), was decreased in diagnostic BM samples. The second one, corresponding to CD14^dim^CD16^+^CD115^+^ non-classical monocytes, was instead increased at diagnosis. Interestingly, moreover, the non-classical monocytes clusters were increased in the BM of relapsed cases when compared to the BM of patients undergoing remission.

**Figure 1:**
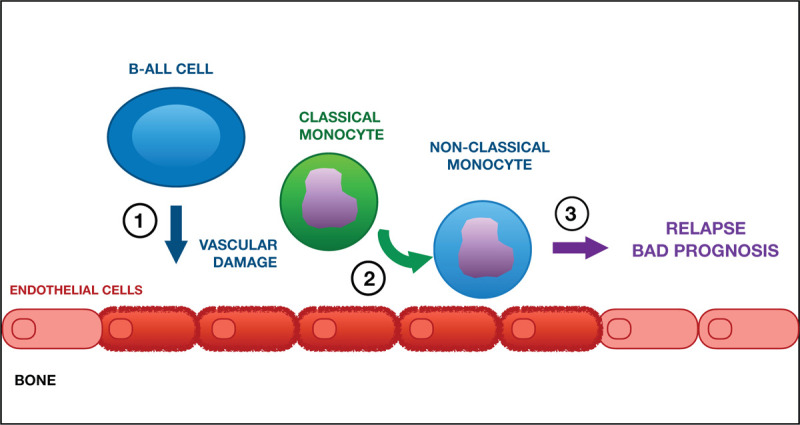
According to the model proposed by Witkowski et al, B-ALL cells, by inducing a vascular damage (1), induce the emergency of non-classical monocytes (2) which facilitate leukemia progression and relapse (3). High monocytes abundance in this model is a negative prognostic factor in both adult and pediatric patients.

Whereas both classical and non-classical monocytes subsets possess antigen processing capacities, the non-classical monocytes (or patrolling monocytes) possess a distinct transcriptomic and metabolic profile and they act mainly as housekeepers of the vascular tissue, where they recognize and clear dying endothelial cells.^3^ Consistently, when compared to their healthy counterpart, the non-classical monocytes identified in diagnostic bone marrow samples showed increased expression of genes involved in monocyte interactions with vascular endothelium during vascular endothelial repair and inflammation. Given that the endothelium plays an important role in promoting the differentiation of classical monocytes into the non-classical ones, the authors hypothesized a model whereby B-ALL cells would induce the emergency of non-classical monocytes by inducing their differentiation from classical monocytes in response to a leukemia-induced vascular damage (Fig. 1). While most non-classical monocytes are believed to derive from classical monocytes, non-classical monocytes might also develop without passing through a classical monocyte stage.^4^,^5^ As such it is not possible to exclude that non-classical monocytes might emerge in B-ALL BM via additional and/or other mechanisms, which are worth to be further investigated, especially in a context whereby the effects of B-ALL cells on the BM vasculature remain largely elusive.

What is the exact role of non-classical monocytes in B-ALL pathogenesis? The authors addressed this point using a well-characterized syngeneic murine model of pediatric *Ph* ^+^ B-ALL, which they show to recapitulate the emergency of a CD11b^+^CX3CR1^+^Ly6C^−^ cell population reminiscent of the non-classical monocytes identified in B-ALL patients. Notably, in this model, the delivery of anti-CSF1R antibodies, which blocked both classical and non-classical monocytes, increased the overall survival of leukemia-transplanted mice concomitantly receiving nilotinib. In addition, whereas the majority of mice succumbed from disease re-emergency following treatment with nilotinib alone, the combination of this drug and anti-CSF1R antibodies decreased the percentages of mice dying from disease recurrence. In line with this, the authors observed a significantly lower overall survival and relapse-free survival in pediatric B-ALL patients presenting with absolute monocytosis at disease diagnosis, independent of other risk factors. Similarly, a high-monocytes abundance predicted inferior overall survival and relapse-free survival in adult B-ALL patients.

Taken together, the findings by Witkowski et al are intriguing and important as relapse remains a major medical challenge especially when it comes to pediatric patients. Yet, a few aspects, which remained unanswered, worth further investigations. How exactly non-classical monocytes promote relapse? Exploring if and how these cells promote the survival of rare dormant B-ALL cells^6^ and/or the clonal evolution of leukemia might provide interesting answers to this important question. Future studies will also be needed to further characterize the cross-talk potentially linking B-ALL blasts, endothelium and monocytes during leukemia progression and to identify the crucial players orchestrating this process. Understanding these issues may provide attractive therapeutic targets for preventing disease progression and /or relapse.
